# Supplementary material for: Yeast Kinesin-5 Motor Protein CIN8 Promotes Accurate Chromosome Segregation
Source: Cells. 2022 Jul 7;11(14):2144. doi: 10.3390/cells11142144 (PMC9316075; doi:10.3390/cells11142144)
Supplement: Supplementary file 1 [file cells-11-02144-s001.zip › cells-1732039-supplementary.pdf]

Table S1. The list of yeast strains used in this study.

Strains	Relevant genotypes	Reference
Y300 (WT)	MAT**a** *ura3-1, his3-11, 15 leu2-3,112 trp1-1, ade2-1, can1-100*	Lab stock
3946-2-3	MAT**a** *cin8-AID-3×Flag::HIS3 ADH1-osTir1-9myc-URA3 PDS1-18myc-LEU2*	This study
3750-3-2	MAT**a** *cin8-AID-3×Flag::HIS3 ADH1-osTir1-9myc-URA3 kip1::KanMX*	This study
YBL063W	MAT**a** *kip1::KanMX*	Lab stock
YEL061C	MAT**a** *cin8::KanMX*	Lab stock
JBY649	MAT**a** *PDS1-18myc-LEU2*	Lab stock
3889-3-1	MAT**a** *cin8-AID-3×Flag::HIS3 ADH1-osTir1-9myc-URA3 kip1::KanMX PDS1-18myc-LEU2*	This study
4232-6-2	MAT**a** *ADH1-osTir1-9myc-URA3 MTW1-GFP-Sphis5⁺ SPC110-mCherry::Hygro*	This study
4232-1-2	MAT**a** *cin8-AID-3×Flag::HIS3 ADH1-osTir1-9myc-URA3 MTW1-GFP-Sphis5⁺ SPC110-mCherry::Hygro*	This study
4232-7-2	MAT**a** *cin8-AID-3×Flag::HIS3 ADH1-osTir1-9myc-URA3 kip1::KanMX MTW1-GFP-Sphis5⁺ SPC110-mCherry::Hygro*	This study
4244-3-4	MAT**a** *ADH1-osTir1-9myc-URA3 promURA3::tetR::GFP-LEU2 CENIV::tetOX448::URA3 SPC110-mCherry::Hygro*	This study
4244-2-1	MAT**a** *cin8-AID-3×Flag::HIS3 ADH1-osTir1-9myc-URA3 promURA3::tetR::GFP-LEU2 CENIV::tetOX448::URA3 SPC110-mCherry::Hygro*	This study
4327-9-2	MAT**a** *promURA3::tetR::GFP-LEU2 CENIV::tetOX448::URA3 SPC110-mCherry::Hygro mad1::HIS3*	This study
4236-1-3	MAT**a** *cin8-AID-3×Flag::HIS3 ADH1-osTir1-9myc-URA3 promURA3::tetR::GFP-LEU2 CENIV::tetOX448::URA3 SPC110-mCherry::Hygro mad1::HIS3*	This study
4003-1-3	MAT**a** *cin8-AID-×3Flag::HIS3 ADH1-osTir1-9myc-URA3 kip1::KanMX mad1::HIS3 PDS1-18myc-LEU2*	This study
771-4-1	MAT**a** *mad1::HIS3 PDS1-18myc-LEU2*	This study
4260-2-4	MAT**a** *cin8-AID-3×Flag::HIS3 ADH1-osTir1-9myc-URA3 kip1::KanMX promURA3::tetR::GFP-LEU2 CENIV::tetO×448::URA3 SPC110-mCherry::Hygro*	This study
4235-9-3	MAT**a** *cin8-AID-3×Flag::HIS3 ADH1-osTir1-9myc-URA3 kip1::KanMX mad1::HIS3 promURA3::tetR::GFP-LEU2 CENIV::tetOX448::URA3 SPC110-mCherry::Hygro*	This study
4330-7-4	MAT**a** *dam1-3A (S257A S265A S292A)::KanMX promURA3::tetR::GFP-LEU2 CENIV::tetO×448::URA3 SPC110-mCherry::Hygro*	This study
4244-6-3	MAT**α** *cin8-AID-3×Flag::HIS3 ADH1-osTir1-9myc-URA3 dam1-3A (S257A S265A S292A)::KanMX promURA3::tetR::GFP-LEU2 CENIV::tetOX448::URA3 SPC110-mCherry::Hygro*	This study
4332-13-2	MAT**a** *cin8-AID-3×Flag::HIS3 ADH1-osTir1-9myc-URA3 PDS1-18myc-LEU2 dam1-3A (S257A S265A S292A)::KanMX*	This study
2425-7-2	MAT**a** *dam1-3A (S257A S265A S292A)::KanMX PDS1-18myc-LEU2*	Lab stock
4332-5-4	MAT**a** *cin8-AID-3×Flag::HIS3 ADH1-osTir1-9myc-URA3 PDS1-18myc-LEU2*	This study
2715-6-4	MAT**a** *ipl1-321 PDS1-18myc-LEU2*	Lab stock
4333-8-1	MAT**a** *ipl1-321 cin8-AID-3×Flag::HIS3 ADH1-osTir1-9myc-URA3 PDS1-18myc-LEU2*	This study
X3338-8D	MAT**α** *ADH1-osTir1-9myc-URA3*	X. Zhao lab
T1129-3C	MAT**a** *ADH1-osTir1-9myc-URA3*	X. Zhao lab
